# Supplementary material for: Synthesis of Novel Tricyclic N-Acylhydrazones as Tubulin Polymerization Inhibitors
Source: Int J Mol Sci. 2025 Sep 20;26(18):9212. doi: 10.3390/ijms26189212 (PMC12470774; doi:10.3390/ijms26189212)
Supplement: Supplementary file 1 [file ijms-26-09212-s001.zip › ijms-3787280-supplementary.pdf]

International Journal of Molecular Sciences

MDPI

Supplementary Material

Synthesis of novel tricyclic *N*-acylhydrazones as tubulin polymerization inhibitors

Paola Corona [1]*, Michele Lai [2], Roberta Ibba [1], Giulia Sciandrone [3], Ilenia Lupinu [4], Battistina Asproni [1], Sandra Piras [1], Antonio Carta [1] and Gabriele Murineddu [1]*

1 Department of Medicine, Surgery and Pharmacy, University of Sassari, 07100 Sassari, Italy; asproni@uniss.it (B.A.); robertaibbaphd@gmail.com (R.I.); piras@uniss.it (S.P.); acarta@uniss.it (A.C.)
2 Department of Translational Medicine and New Technologies in Medicine and Surgery, Retrovirus Centre, University of Pisa, 56127 Pisa, Italy; michele.lai@unipi.it
3 Department of Medical Biotechnologies, University of Siena, 53100 Siena, Italy; g.sciandrone@student.unisi.it
4 Department of Chemical, Physical, Mathematical and Natural Sciences, University of Sassari, 07100 Sassari, Italy; i.lupinu@phd.uniss.it
* Correspondence: pcorona@uniss.it (P.C.); muri@uniss.it (G.M.)

Contents

Figure. S1 – S2. NOESY spectra of compounds **1a – 2a**

Figure. S3 – S18. ^{1}H and ^{13}C NMR spectra of compounds **1a – j** and **2a – d**,**i** and **j**

Figure. S1. NOESY spectrum of compound **1a**

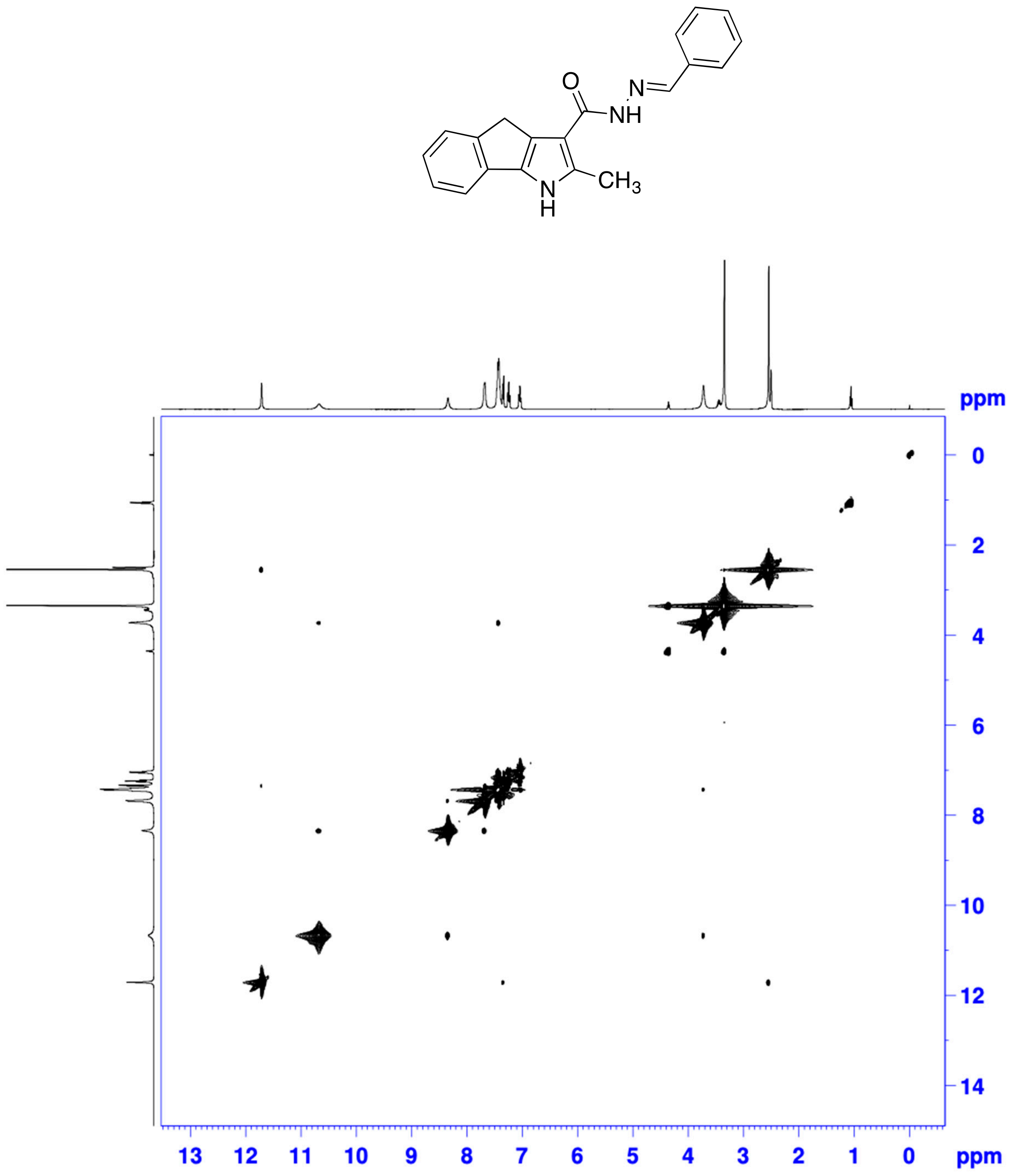

Figure. S2. NOESY spectrum of compound **2a**

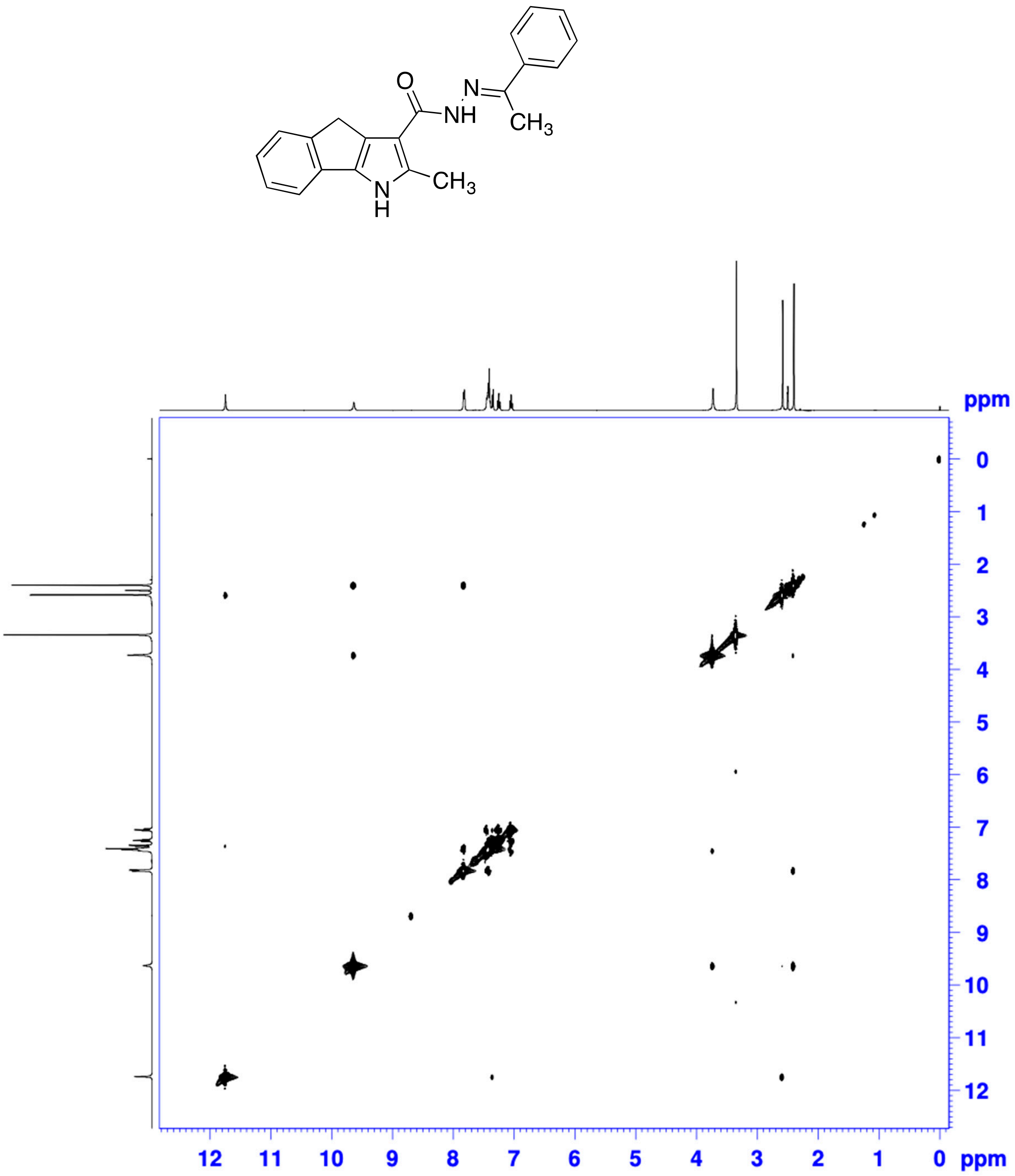

Figure. S3. ^{1}H and ^{13}CNMR spectra of compound **1a**

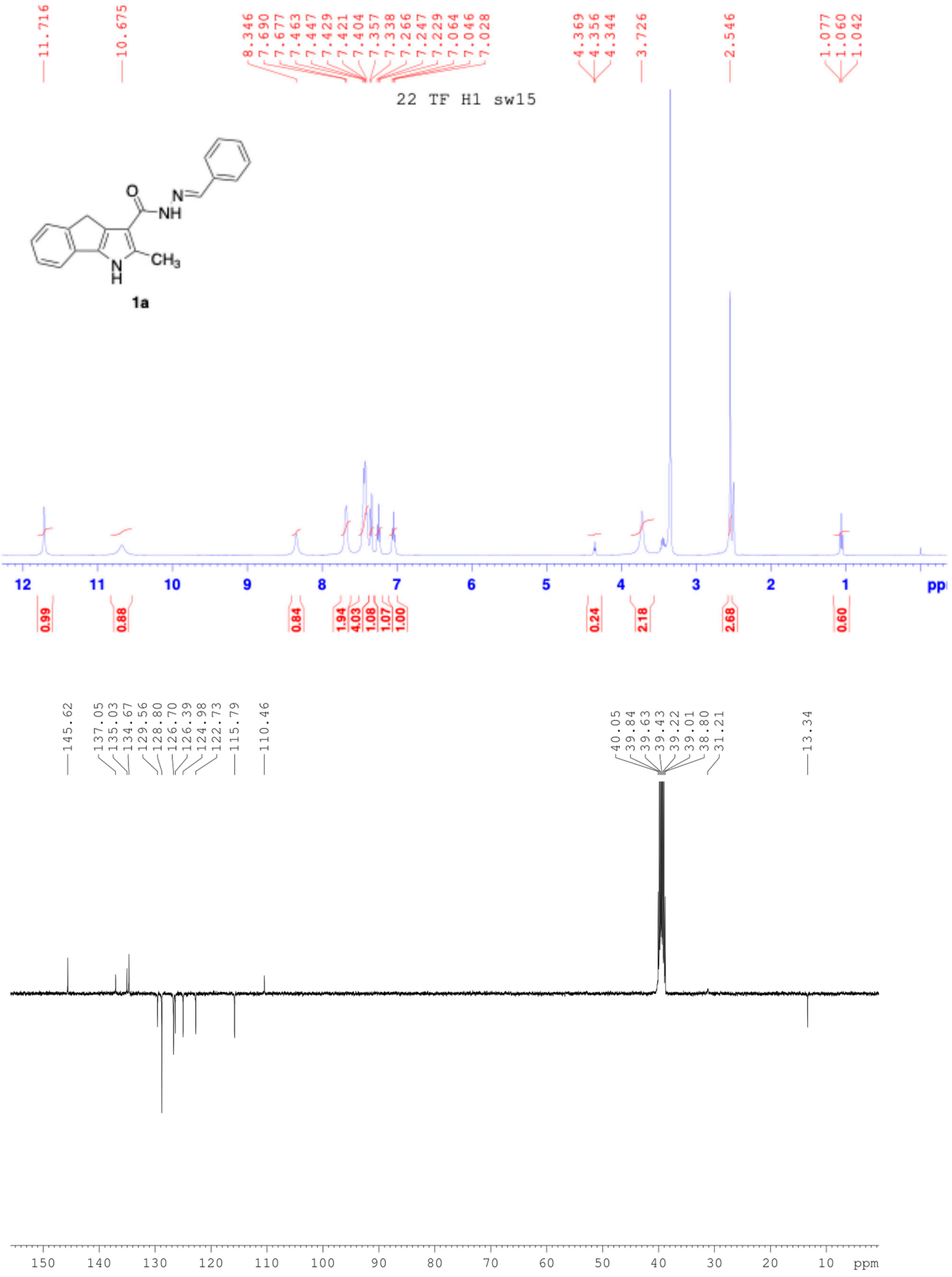

Figureure S4. ^{1}H and ^{13}C NMR spectra of compound **1b.**

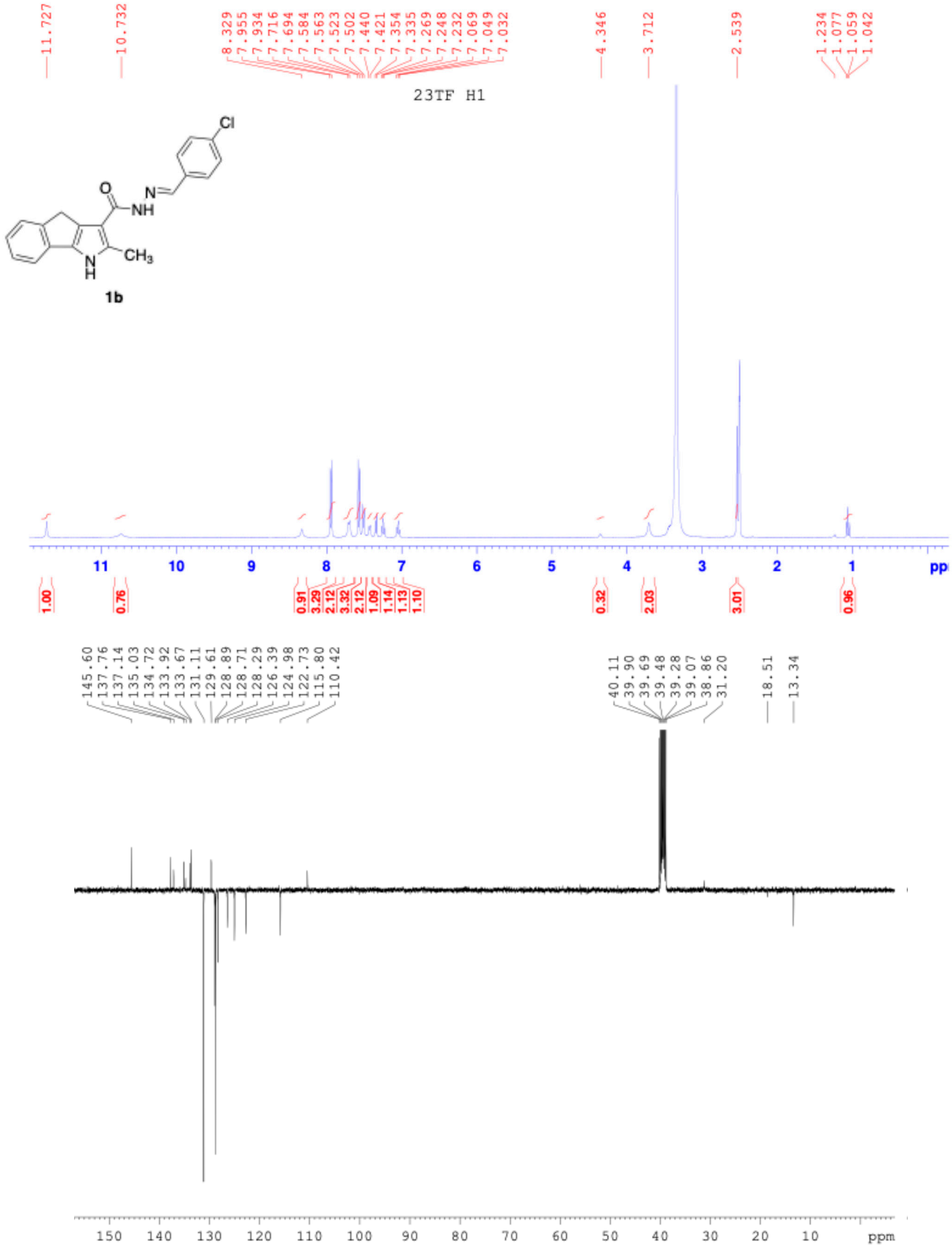

Figure. S5. ^{1}H and ^{13}C NMR spectra of compound **1c**

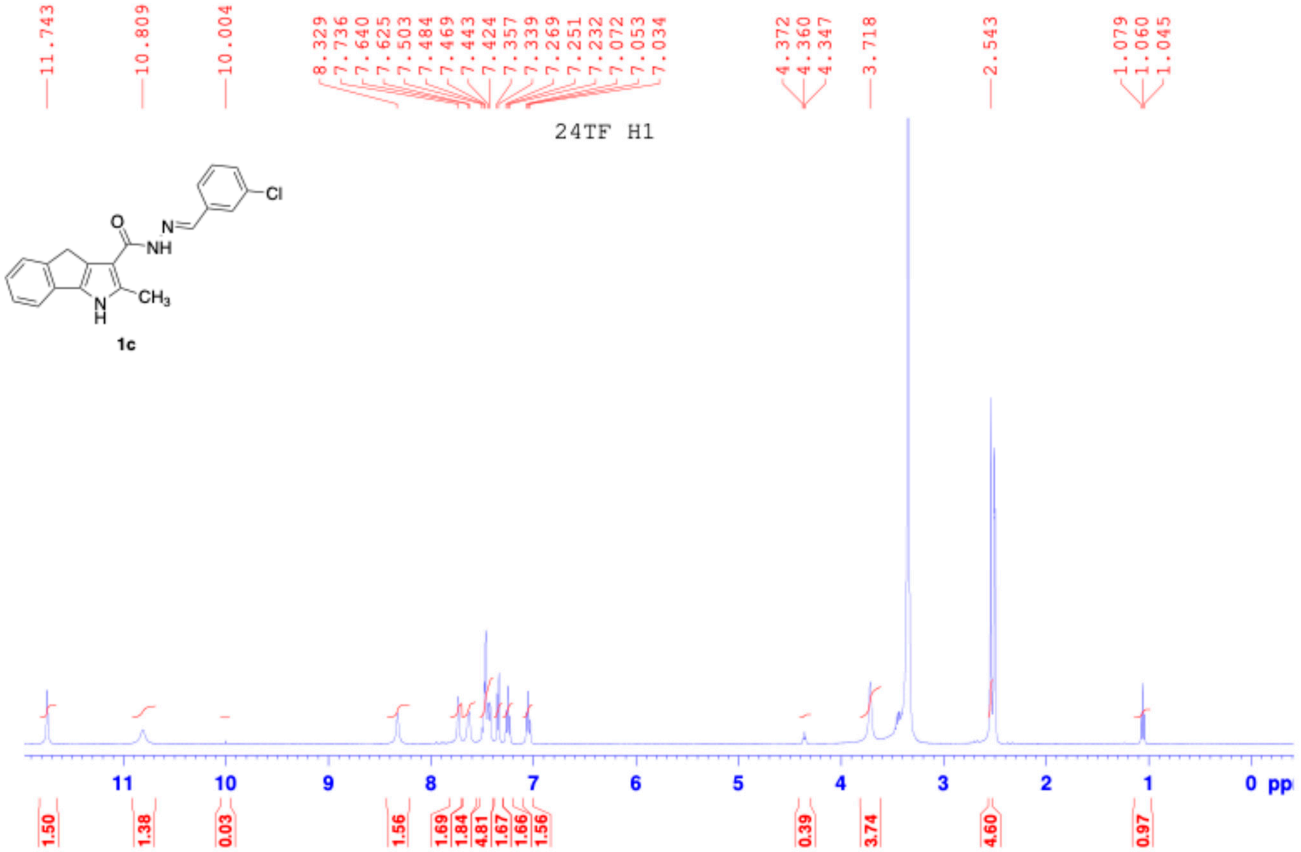

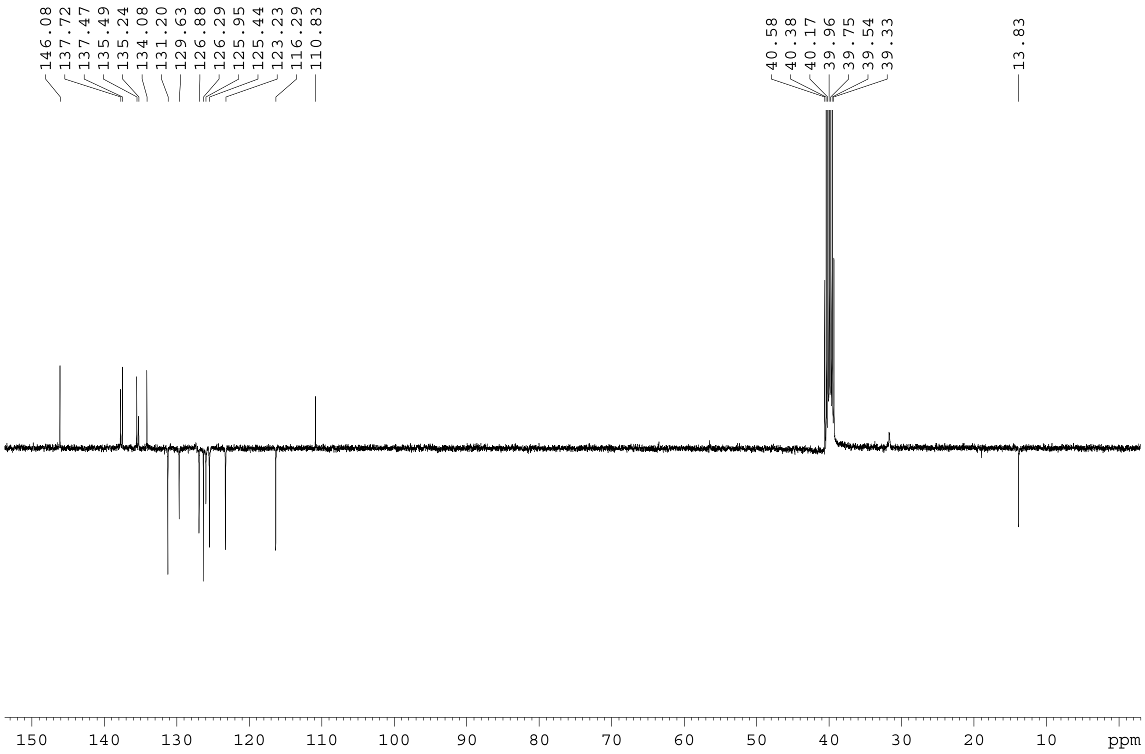

Figure. S6. ^{1}H and ^{13}C NMR spectra of compound **1d**

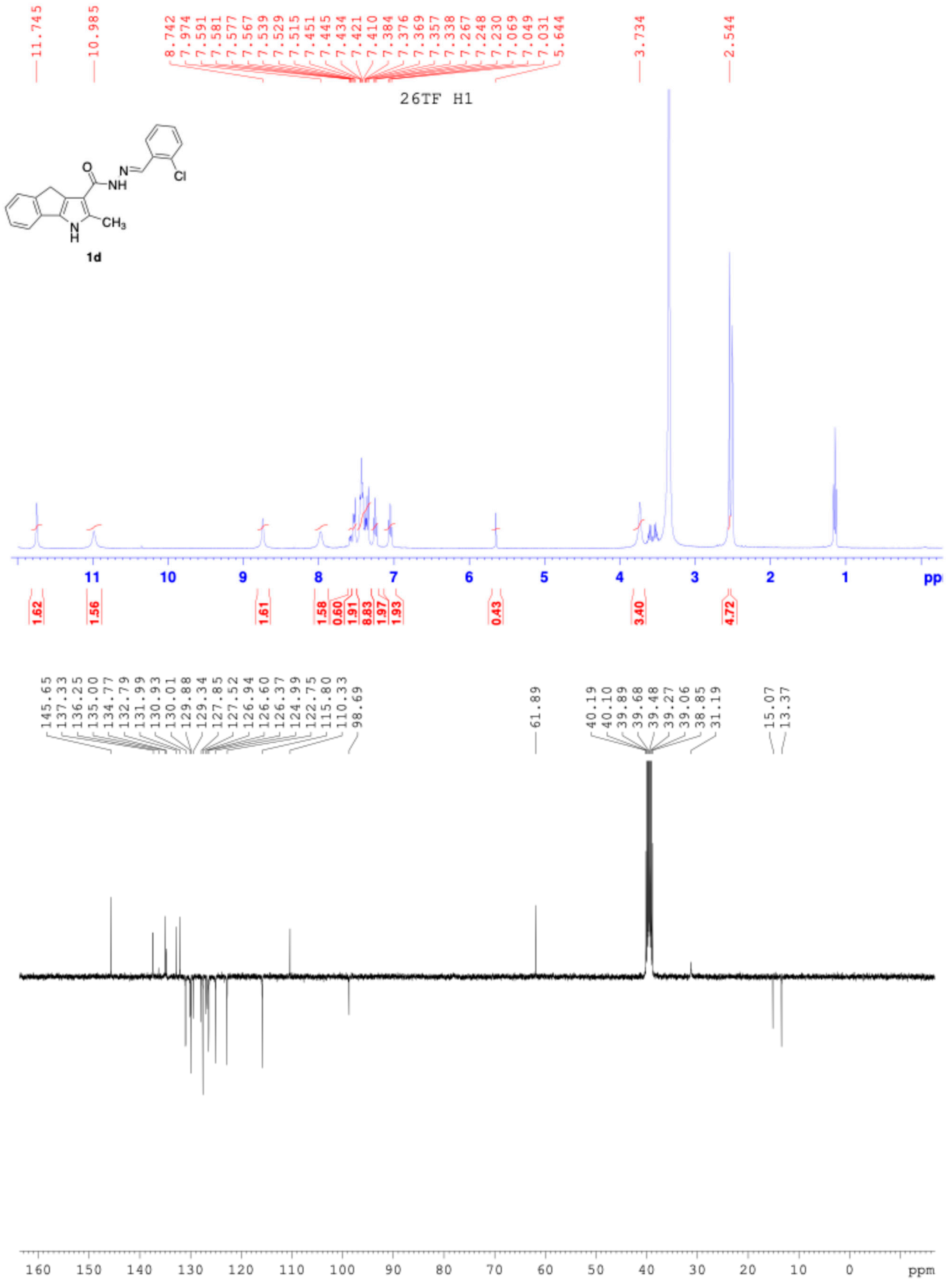

Figure. S7. ^{1}H and ^{13}C NMR spectra of compound **1e**

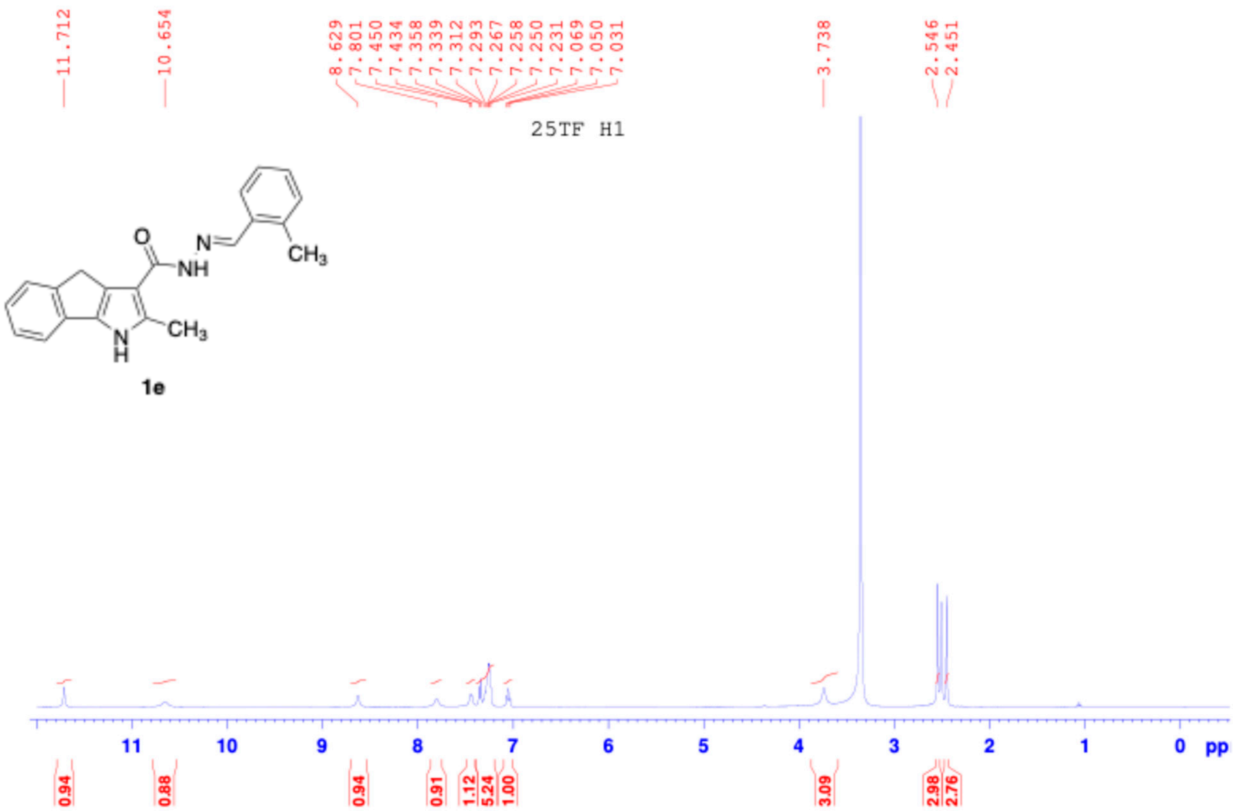

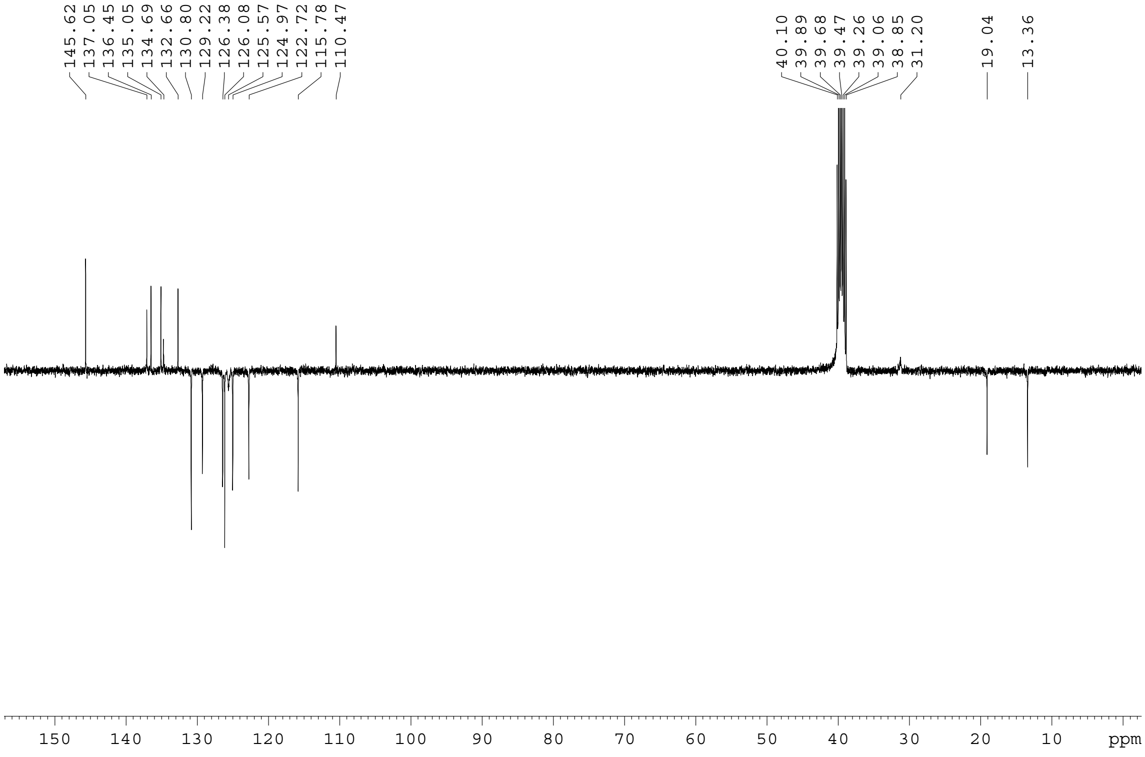

Figure. S8. ^{1}H and ^{13}C NMR spectra of compound **1f**

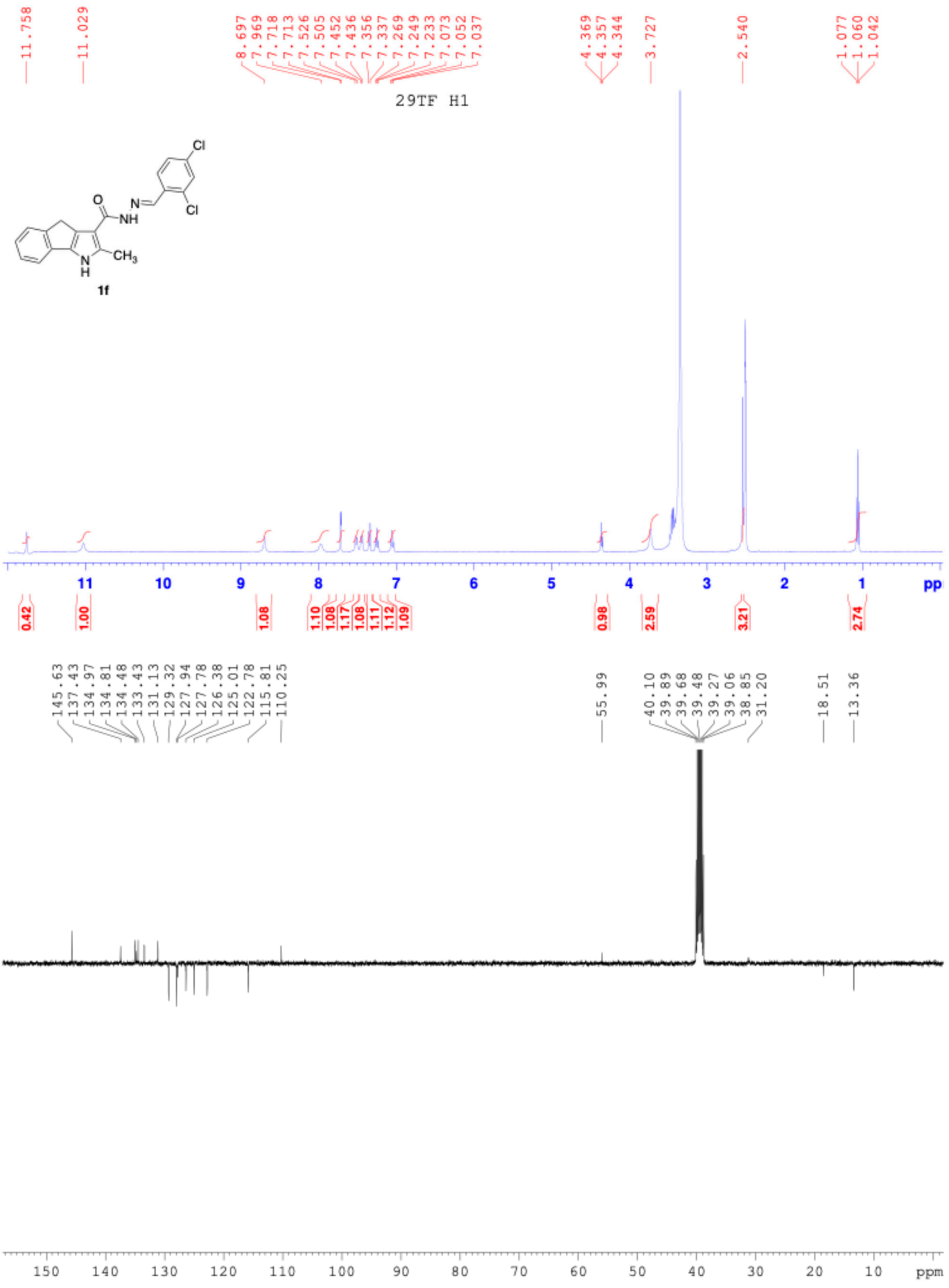

Figure. S9. ^{1}H and ^{13}C NMR spectra of compound **1g**

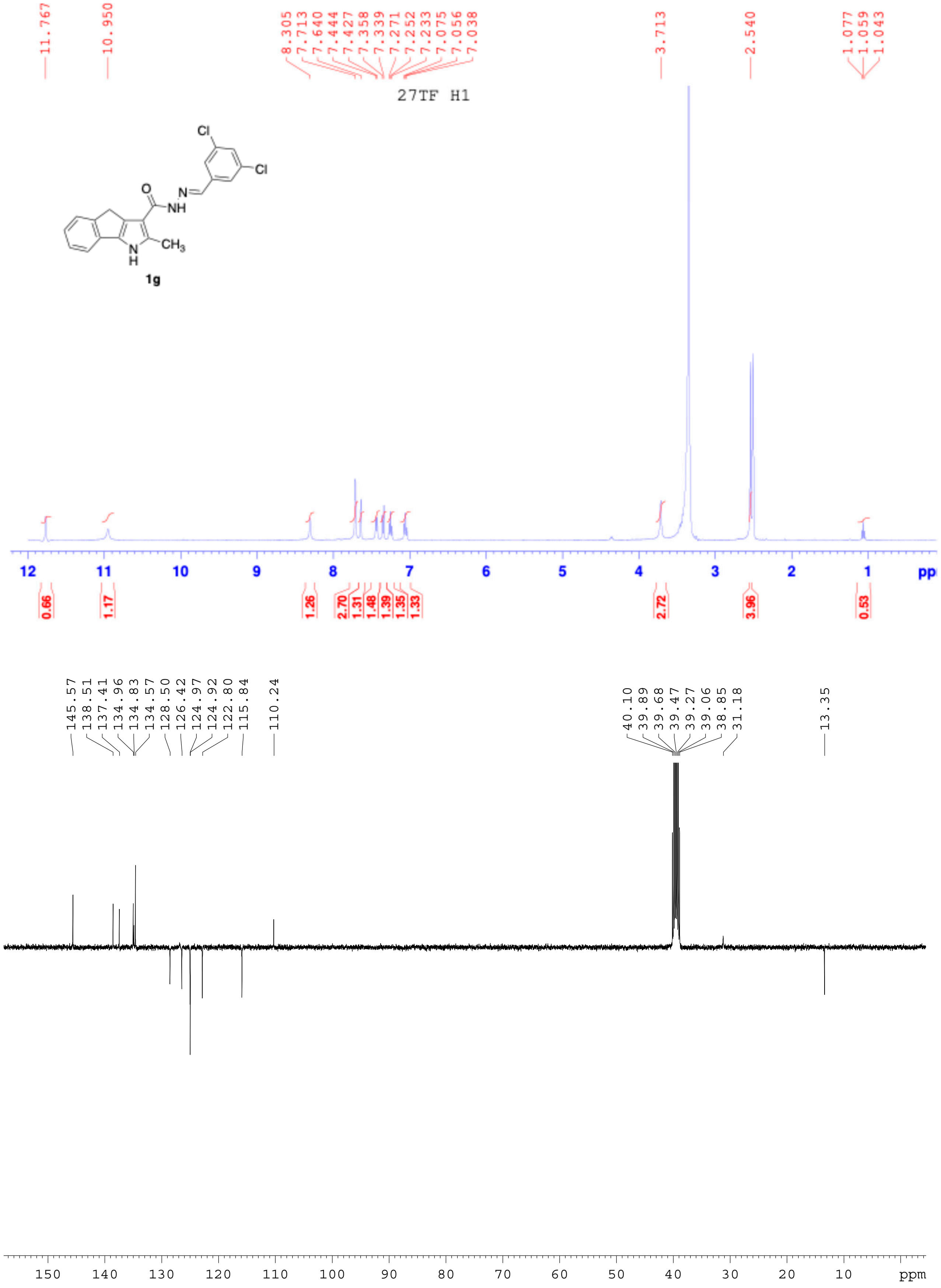

Figure. S10. ^{1}H and ^{13}C NMR spectra of compound **1h**

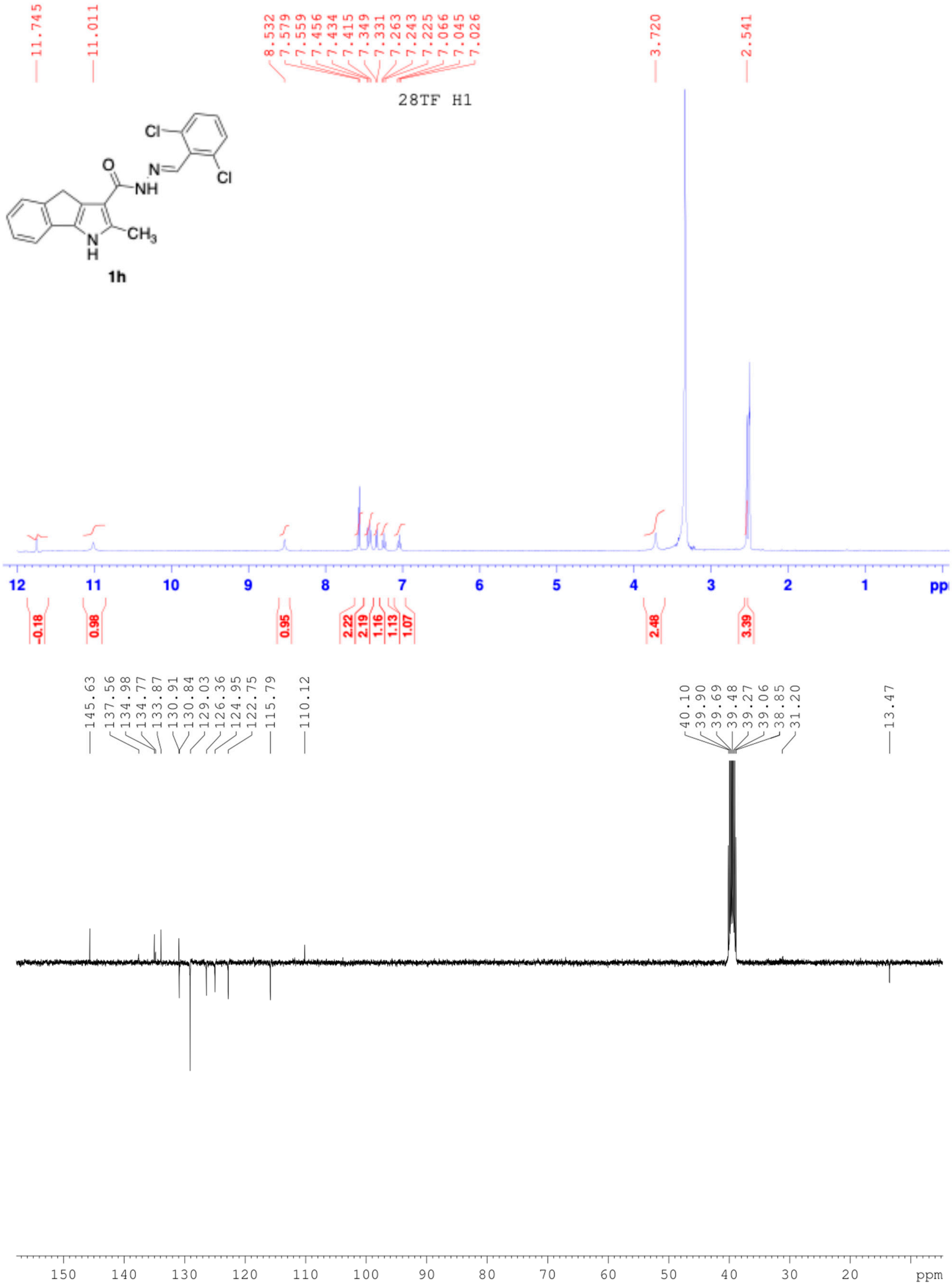

Figure. S11. ¹H and ¹³C NMR spectra of compound **1i**

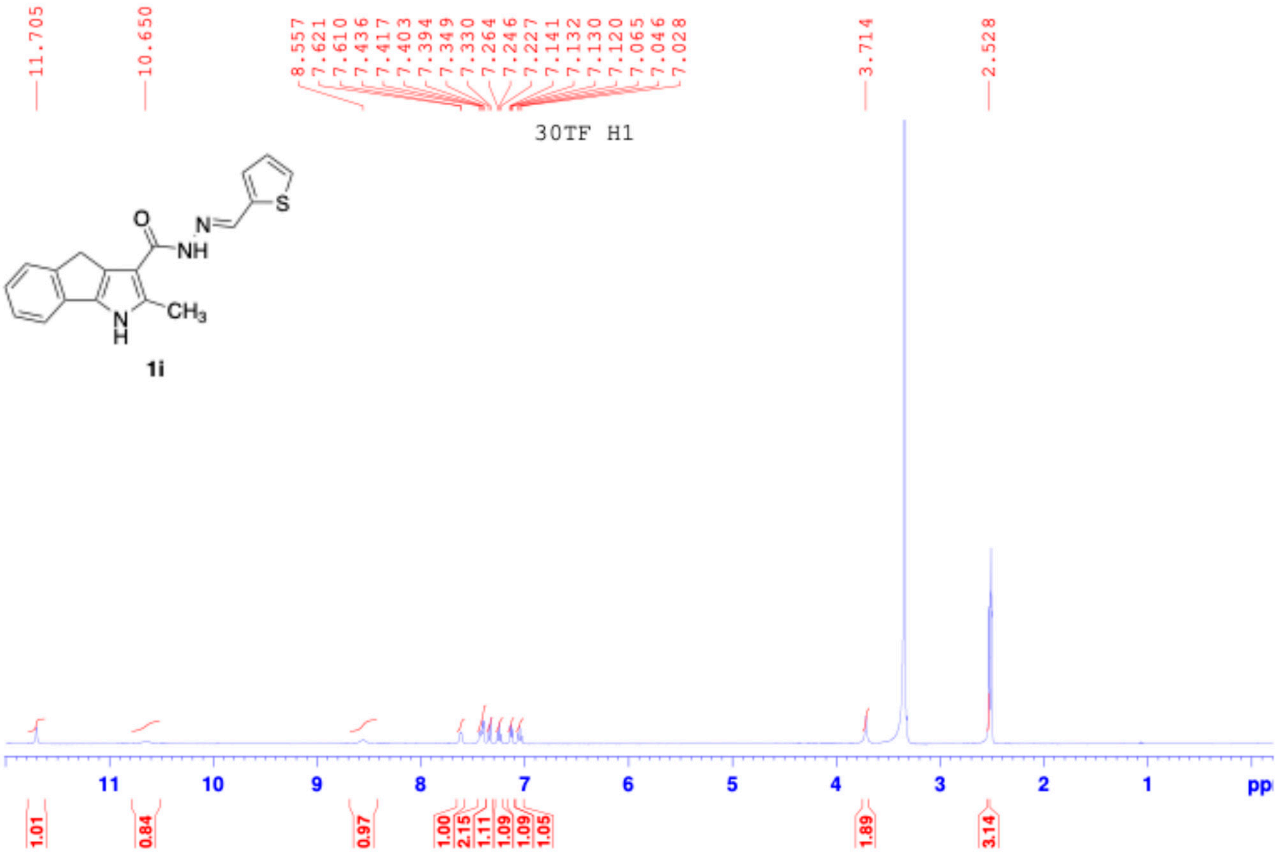

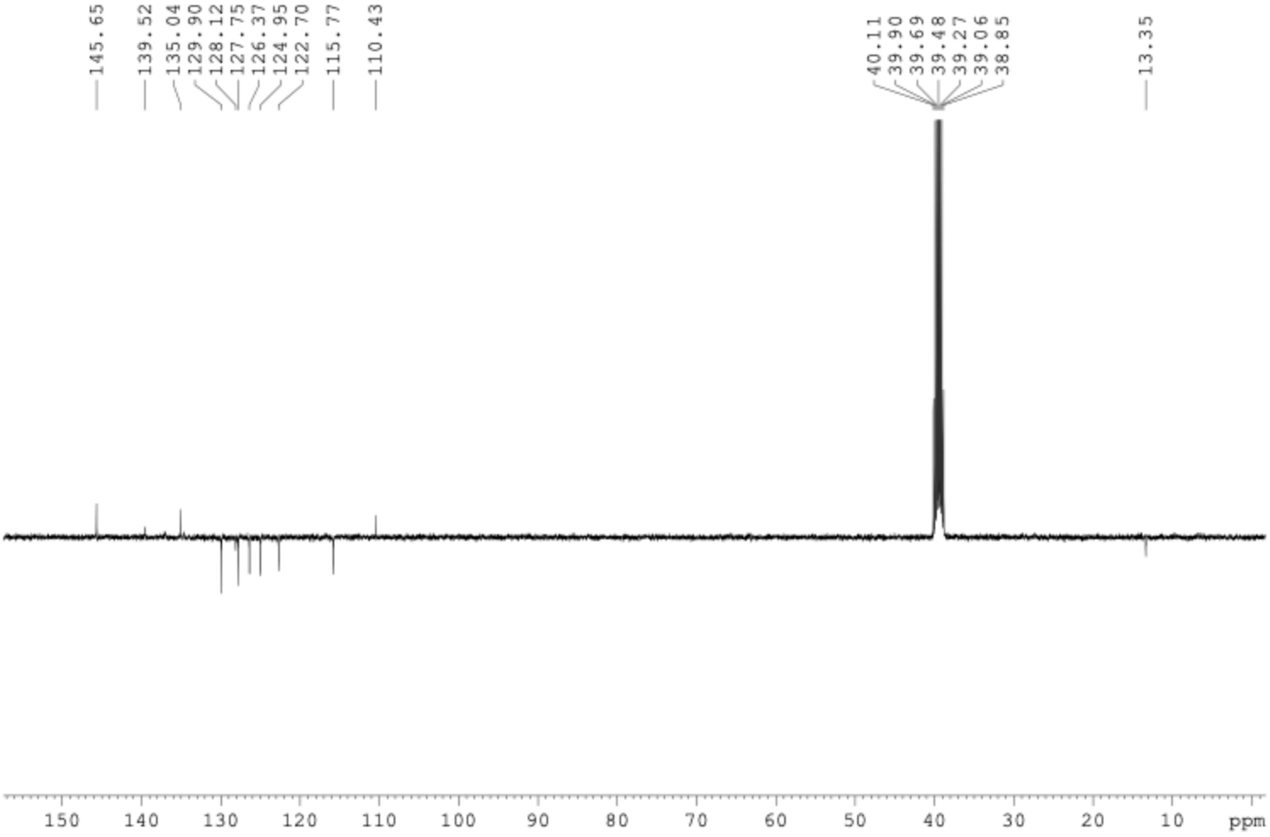

Figure. S12. ^{1}H and ^{13}C NMR spectra of compound **1j**

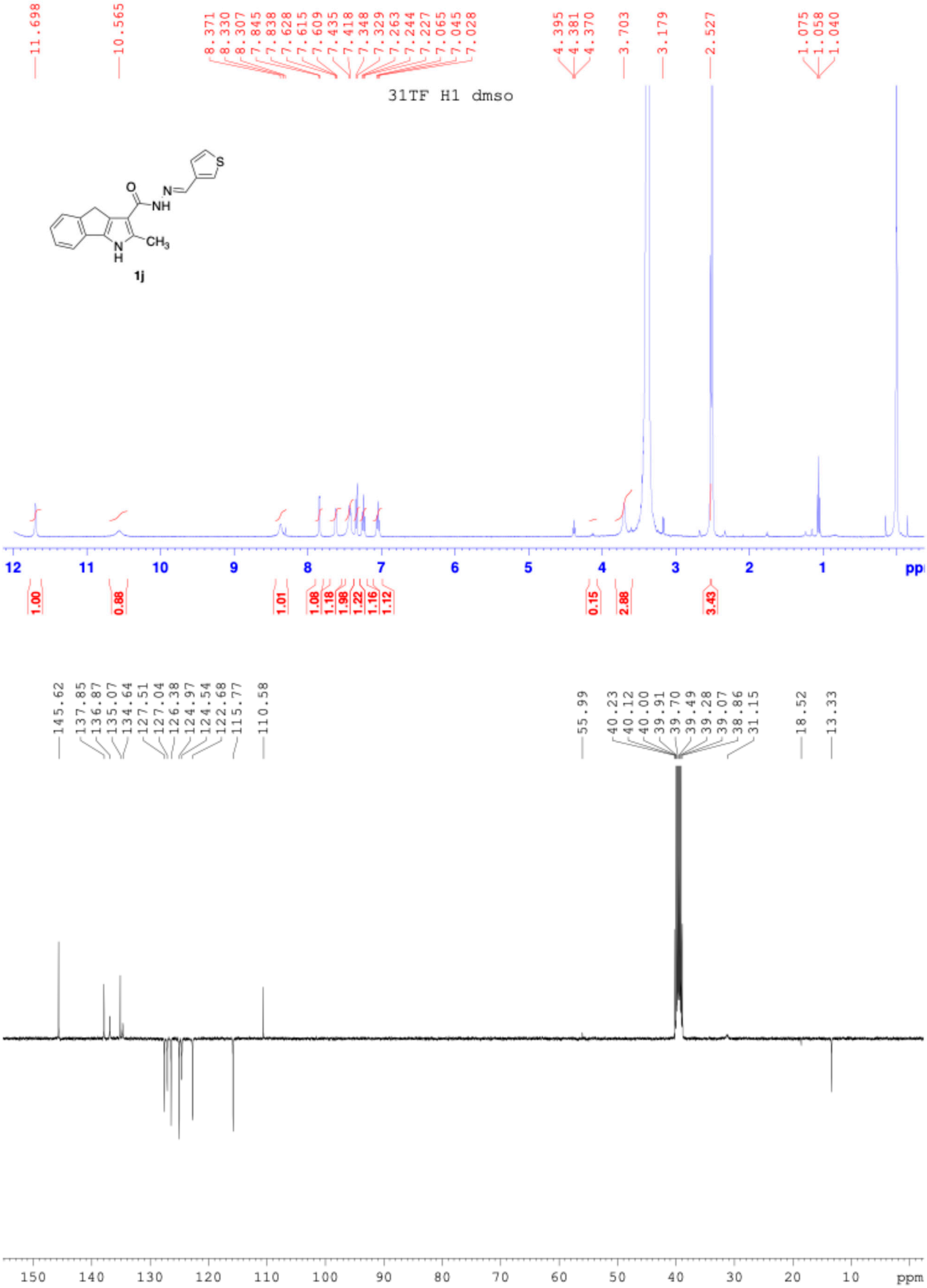

Figure. S13. ^{1}H and ^{13}C NMR spectra of compound **2a**

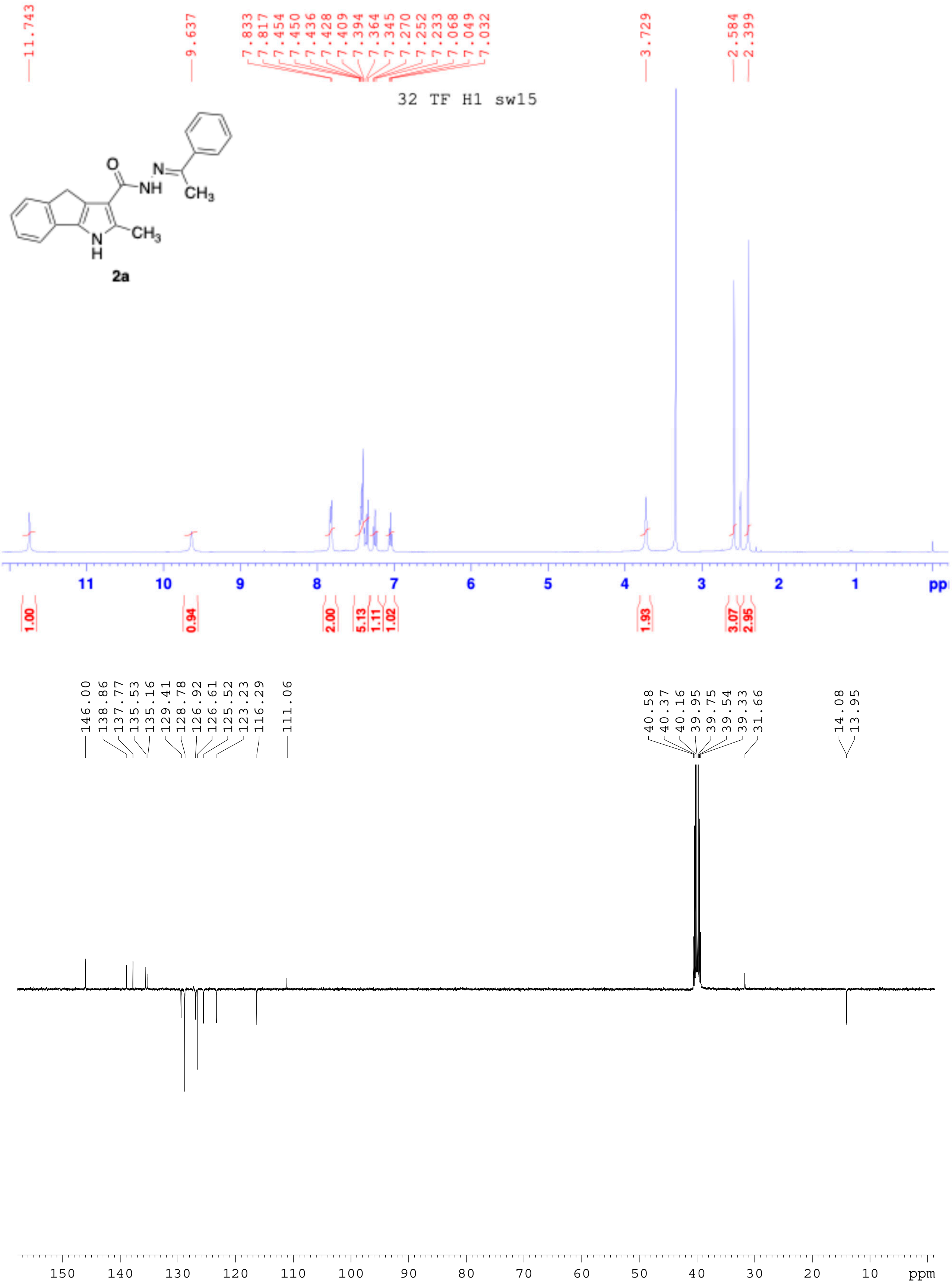

Figure. S14. ^{1}H and ^{13}C NMR spectra of compound **2b**

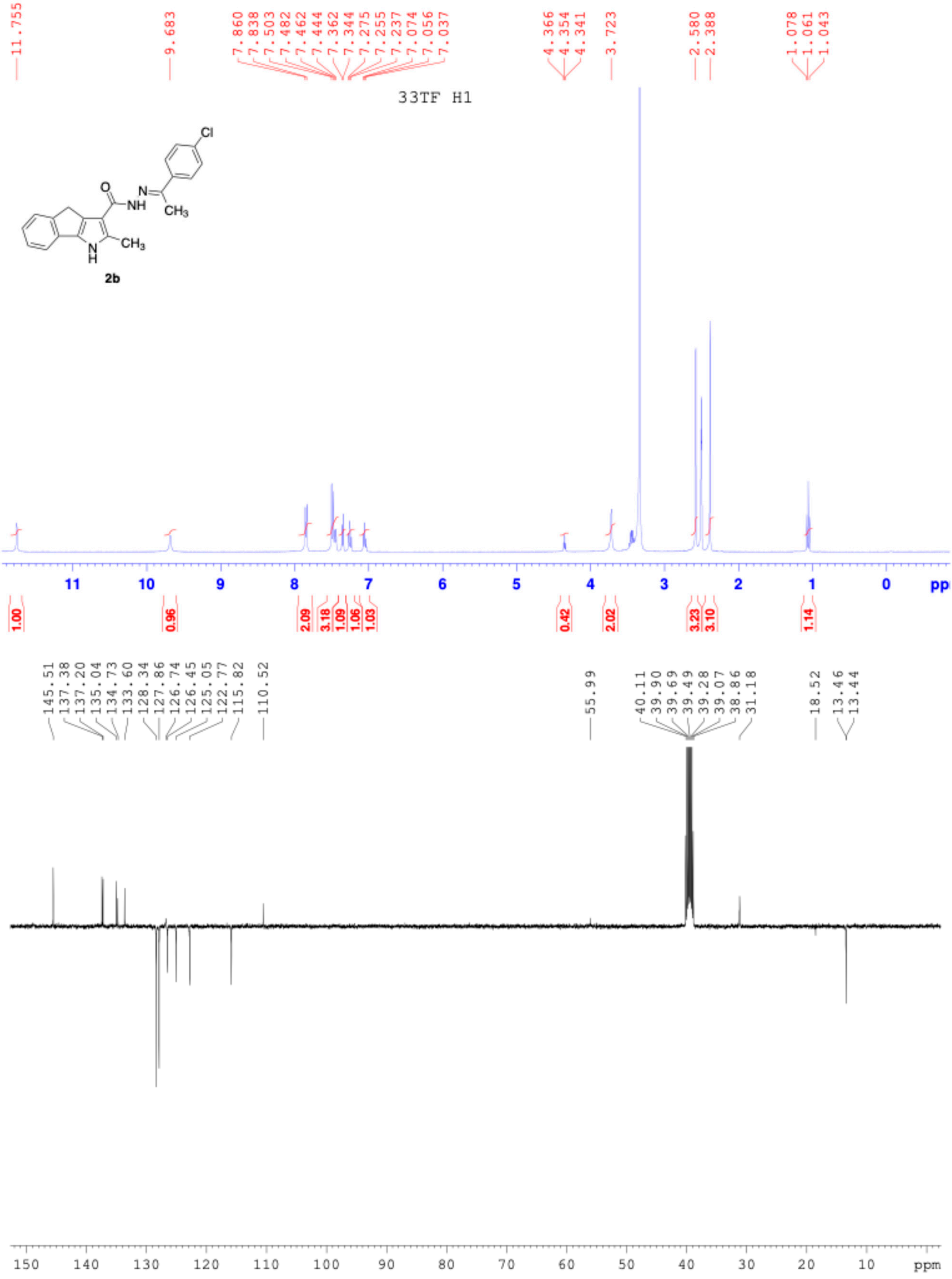

Figure. S15. ^{1}H and ^{13}C NMR spectra of compound **2c**

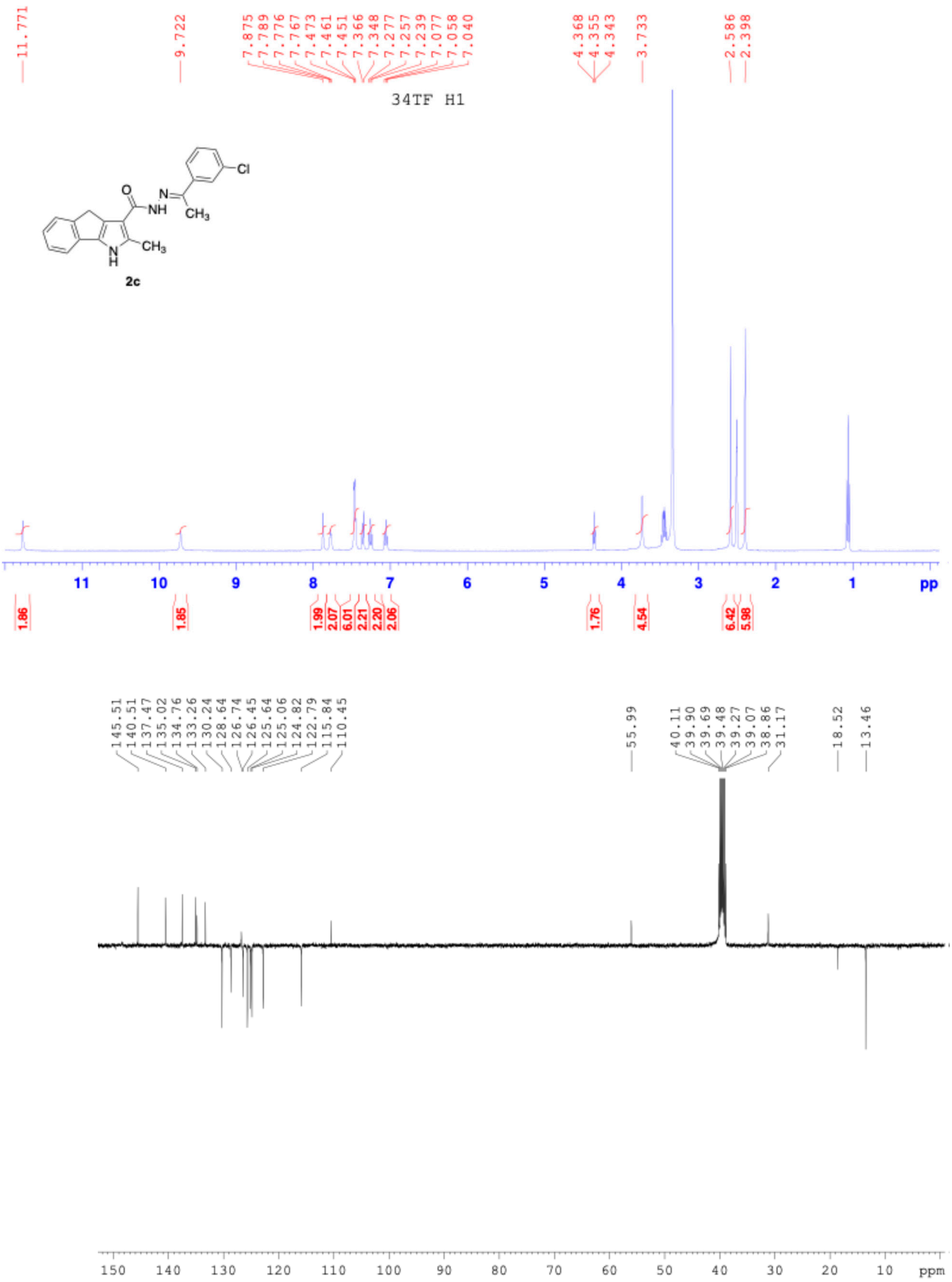

Figure. S16. ^{1}H and ^{13}C NMR spectra of compound **2d**

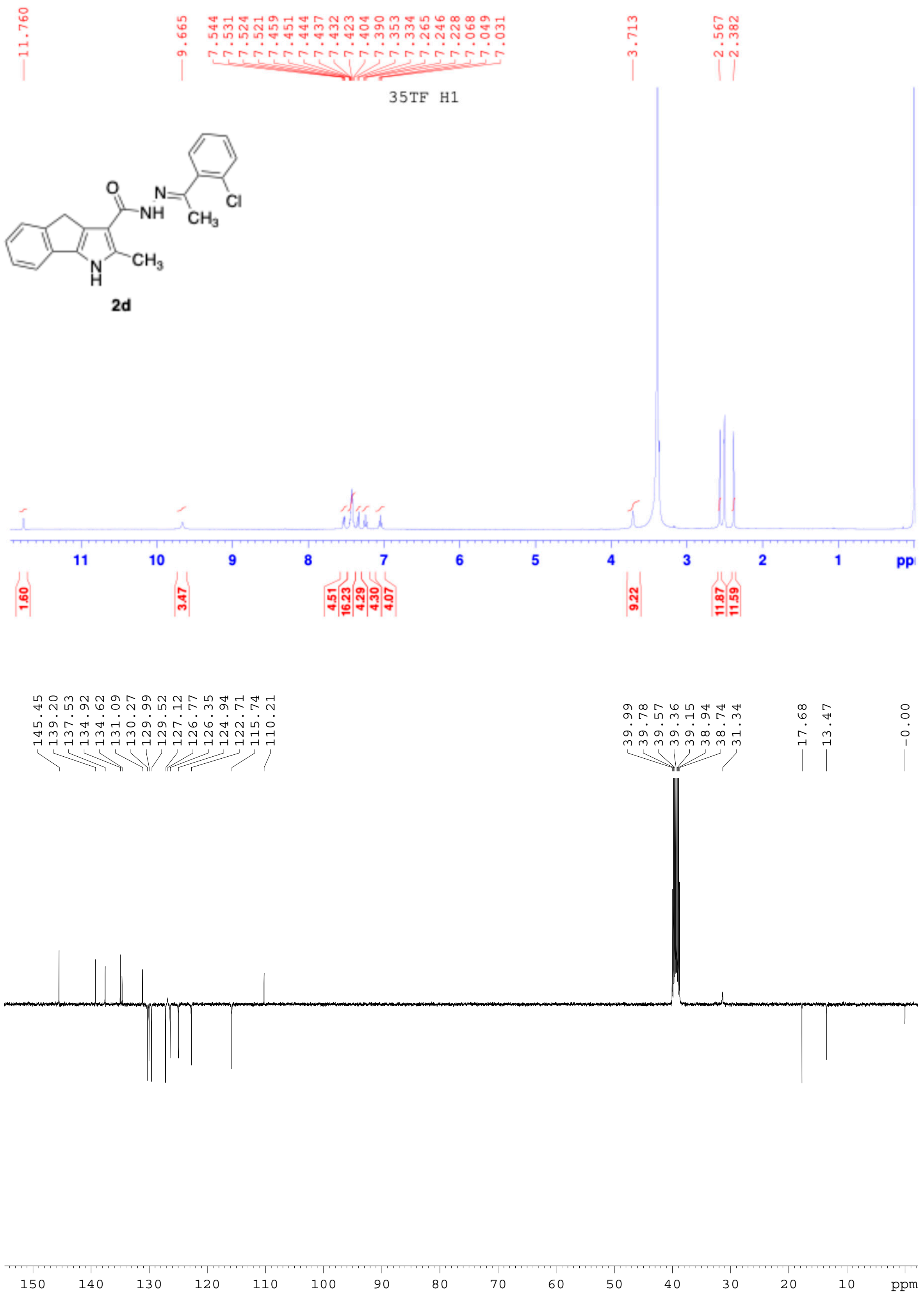

Figure. S17. ^{1}H and ^{13}C NMR spectra of compound **2i**

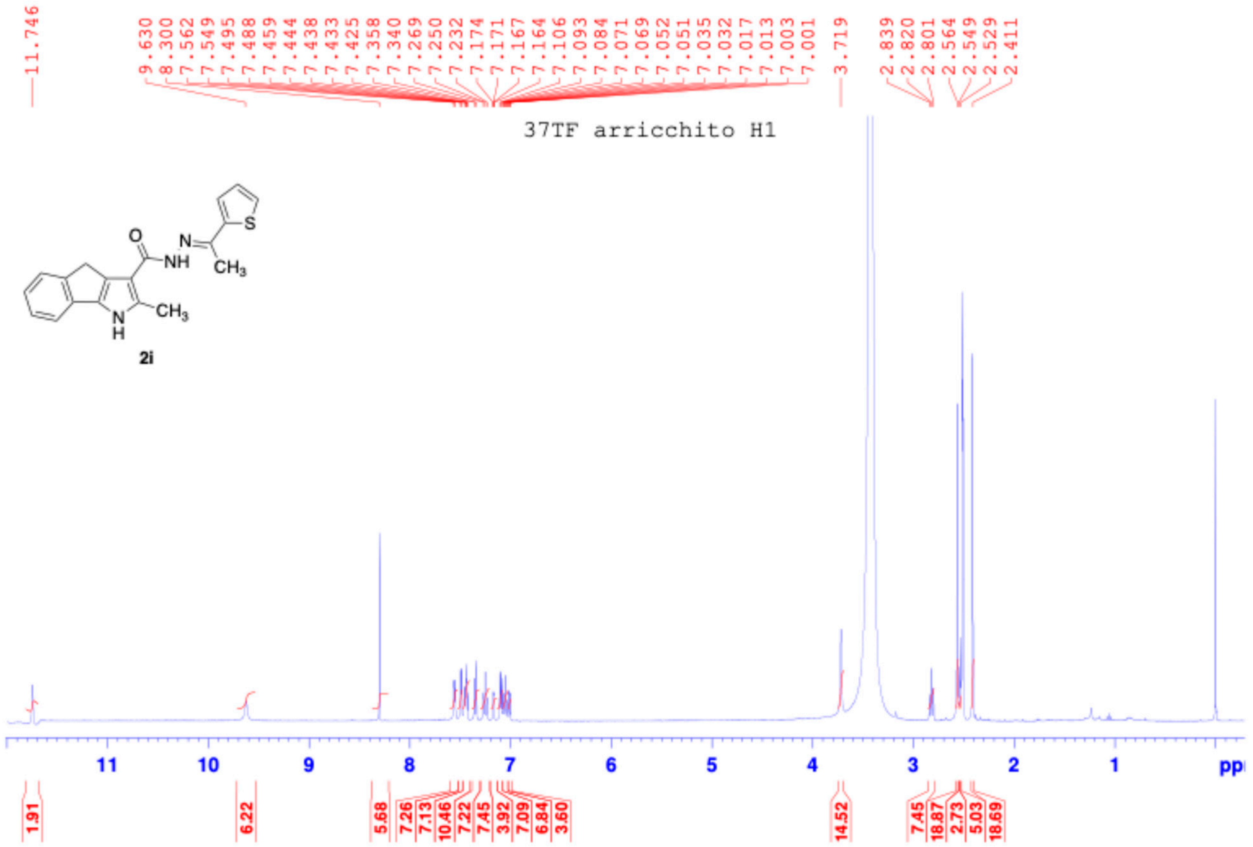

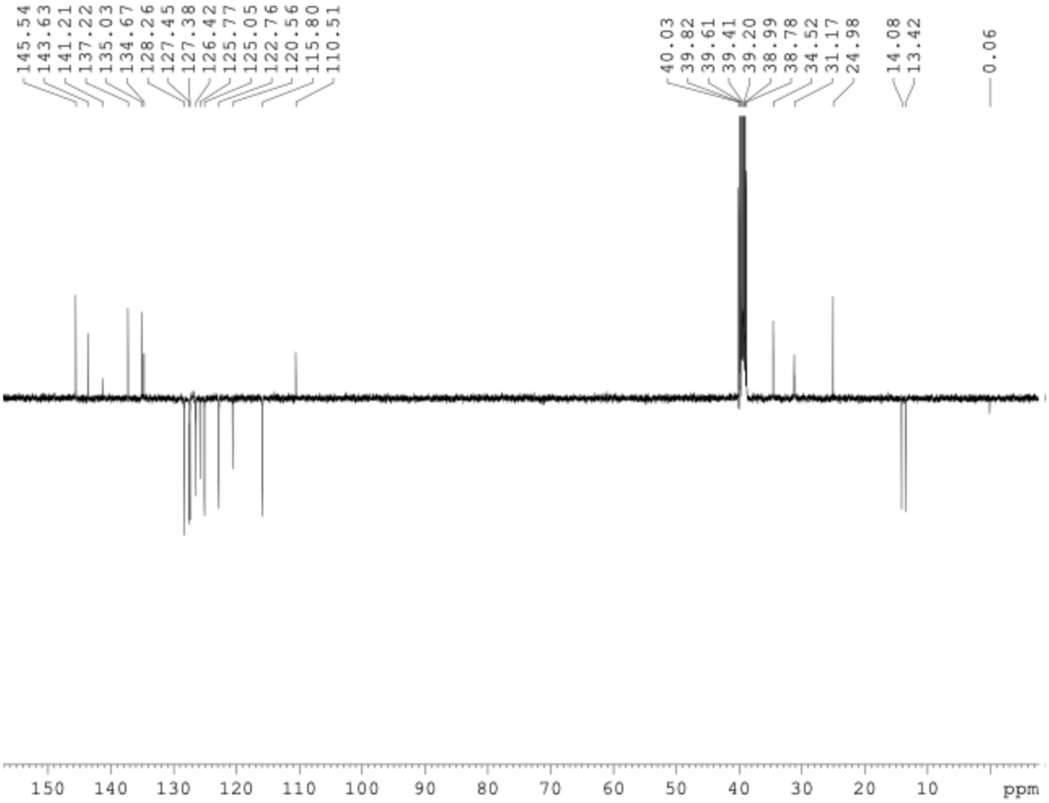

Figure. S18. ^{1}H and ^{13}C NMR spectra of compound **2j**

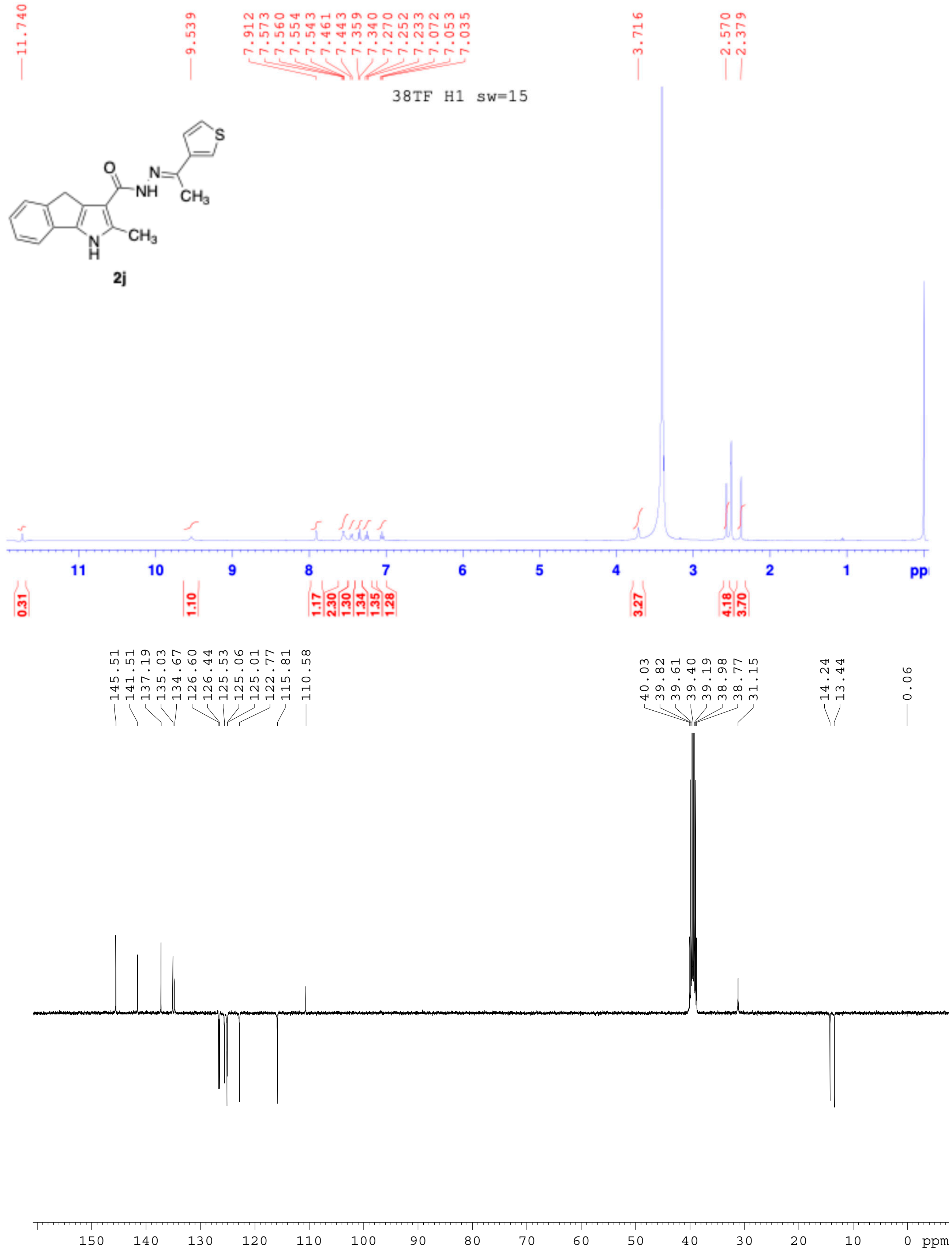

Disclaimer/Publisher's Note: The statements, opinions and data contained in all publications are solely those of the individual author(s) and contributor(s) and not of MDPI and/or the editor(s). MDPI and/or the editor(s) disclaim responsibility for any injury to people or property resulting from any ideas, methods, instructions or products referred to in the content.
